# Editorial: Complementary and alternative therapy for pain disorders: from bench to clinical practice

**DOI:** 10.3389/fneur.2024.1498525

**Published:** 2024-12-05

**Authors:** Qinhong Zhang, Shiyan Yan, Michael Furian, Jinhuan Yue, Xiaoling Li, Hao Chi, Hui-Tzu Yang, David M. Zheng, Tiancheng Xu, Brenda Golianu, Guanhu Yang

**Affiliations:** ^1^Heilongjiang University of Chinese Medicine, Harbin, China; ^2^Shenzhen Frontiers in Chinese Medicine Research Co., Ltd., Shenzhen, China; ^3^International Acupuncture and Moxibustion Innovation Institute, School of Acupuncture-Moxibustion and Tuina, Beijing University of Chinese Medicine, Beijing, China; ^4^Research Department, Swiss TCM University, Bad Zurzach, Switzerland; ^5^Division of CT and MRI, First Affiliated Hospital of Heilongjiang University of Chinese Medicine, Harbin, China; ^6^Clinical Medical College, Southwest Medical University, Luzhou, China; ^7^Faculty of Chinese Medicine, Macau University of Science and Technology, Cotai, Macau SAR, China; ^8^Key Laboratory of Acupuncture and Medicine Research of Ministry of Education, Nanjing University of Chinese Medicine, Nanjing, China; ^9^Department of Anesthesia, Stanford University School of Medicine, Palo Alto, CA, United States; ^10^Department of Specialty Medicine, Ohio University, Athens, OH, United States

**Keywords:** pain, complementary and alternative therapy, acupuncture, yoga, cupping, mechanism, efficacy, safety

## 1 Introduction

Pain disorders are a widespread clinical concern, affecting millions globally and leading to diminished quality of life and reduced productivity (1). While pharmacological treatments remain a cornerstone in pain management, they often present challenges, such as adverse side effects, drug tolerance, and insufficient relief for certain patients (2). These limitations have led to a growing exploration of complementary and alternative therapies (CATs) as non-pharmacological solutions for pain (3). This editorial consists of 17 recent studies authored by 149 researchers from five countries, highlighting the role of CATs—including acupuncture, yoga, and cupping—in addressing pain disorders. The discussion covers key aspects of CATs, such as their underlying mechanisms, clinical efficacy, and integration into conventional medical practice.

## 2 Mechanistic insights into complementary and alternative therapies

Understanding the underlying mechanisms of CATs is essential for their broader acceptance in clinical practice. Huang et al. investigated acupuncture's effects on chronic spontaneous urticaria using functional MRI, identifying alterations in brain network function that may underlie its therapeutic effects. Similarly, Ye et al. explored the role of astrocyte activation in the somatosensory cortex as a mechanism for electroacupuncture's analgesic effects in acid-induced pain, providing a novel insight into how acupuncture may modulate pain pathways at the neurobiological level.

## 3 Evaluating the efficacy and safety of CATs across pain conditions

Several studies have assessed the efficacy of various CATs across different pain conditions, highlighting both potential benefits and areas where further research is needed. Wang L. et al. performed an evidence-mapping study on cupping therapy, which suggested that cupping may have beneficial effects on pain, although the overall quality of evidence was varied, ranging from low to moderate. Ko and Kim investigated the impact of acupuncture on pain and substance P levels in middle-aged women with chronic neck pain, demonstrating significant pain reduction and highlighting acupuncture's potential role in modulating neuropeptides associated with pain perception. Additionally, Zhang, Chang et al. reviewed systematic reviews on yoga for chronic low back pain, finding that yoga can be an effective and safe intervention, though the quality of the evidence was inconsistent.

## 4 Clinical applications of CATs in specific populations

Research has also focused on the application of CATs in specific patient populations, enhancing our understanding of their practical use in clinical settings. Zhang, Chen et al. studied auricular acupuncture as an adjunct for postoperative pain management in patients undergoing total knee arthroplasty (TKA). Their findings indicated significant reductions in postoperative pain and inflammation, supporting the use of acupuncture as a complementary therapy in surgical recovery. Liu et al. conducted a network meta-analysis to compare various acupuncture modalities combined with multimodal analgesia for post-TKA pain. This study demonstrated that these combined approaches offer superior pain relief and functional outcomes compared to multimodal analgesia alone, suggesting a beneficial role for acupuncture in enhancing postoperative pain management.

The Alberta Complementary Health Integration Project (ABCHIP) provided real-world evidence on the integration of acupuncture in treating pain and mental health concerns in vulnerable populations, such as youth and the elderly. Results indicated significant improvements in pain severity, sleep quality, and mental health measures, underscoring the potential of acupuncture as part of a holistic approach to patient care (Lu et al. (a)).

## 5 Expanding the applications of CATs

Beyond traditional applications, CATs are being explored for their potential benefits in managing complex conditions such as cancer-related pain and neurological disorders. Zhou et al. conducted a systematic review and meta-analysis on acupuncture point stimulation for stomach cancer pain, finding it to be more effective than standard medication-based approaches, thus supporting its use in oncology settings. Additionally, Zhang J. et al. detailed a protocol for a systematic review of fire needle therapy for cancer pain, aiming to clarify its efficacy and safety as an adjunctive treatment for cancer-related pain management. Wang Z. et al. explored the efficacy of acupuncture in managing facial nerve edema in patients with acute Bell's palsy, contributing to the understanding of acupuncture's role in acute neuropathic pain conditions.

## 6 CATs for osteoarthritis and chronic pain management

Osteoarthritis, particularly knee osteoarthritis (KOA), is a common chronic pain condition where CATs have shown potential benefits. Zhao et al. outlined a study protocol to evaluate the effectiveness of acupuncture and tuina in managing KOA, addressing the controversy surrounding their clinical application by providing structured evidence on short- and long-term outcomes. Qiu et al. proposed combining catgut embedding in acupoints with repetitive transcranial magnetic stimulation for treating postmenopausal osteoporosis, offering an innovative approach that merges neurostimulation with traditional therapies to address both pain and bone health.

## 7 Addressing pain and mental health with integrated therapies

The intersection of pain management and mental health is increasingly recognized, with studies exploring how CATs can address both domains simultaneously. Lu et al. (b) from the Alberta Complementary Health Integration Project highlighted the benefits of acupuncture in alleviating pain and improving mental health outcomes in a diverse patient population, demonstrating significant reductions in pain severity, anxiety, and depressive symptoms. Xiong et al.'s systematic review on acupuncture for myofascial pain syndrome confirmed its effectiveness in reducing pain, suggesting the need for further studies to optimize treatment protocols for best results.

## 8 Future directions and global trends in CATs research

Li et al.'s bibliometric analysis provided a comprehensive overview of global trends in acupuncture research for pain management from 2010 to 2023, identifying key areas for future research such as standardization of treatment protocols and enhanced international collaboration. Song et al. reviewed the role of CATs in migraine treatment, detailing various mechanisms through which therapies like acupuncture and herbal treatments may alleviate migraine symptoms, thus expanding the understanding of CATs beyond traditional pain disorders.

## 9 Summary

This editorial synthesizes recent advancements in complementary and alternative therapies for pain disorders, highlighting their potential as effective, non-pharmacological treatment options. By bridging the gap between bench research and clinical application, these studies underscore the importance of continued investigation into CATs to improve their integration into mainstream healthcare. Future research should aim to enhance the quality of evidence, standardize treatment protocols, and foster interdisciplinary collaboration, ultimately leading to more comprehensive, patient-centered pain management strategies.
